# Enhancing Anti-Static Performance of Fibers by Construction of the Hybrid Conductive Network Structure on the Fiber Surface

**DOI:** 10.3390/polym13142248

**Published:** 2021-07-09

**Authors:** Congcong Xu, Lin Fang, Mingming Yu, Musu Ren, Jinliang Sun, Liying Zhang

**Affiliations:** 1 Research Center for Composite Materials, Shanghai University, Shanghai 200444, China; xucongjx@163.com (C.X.); lfang@shu.edu.cn (L.F.); msren@shu.edu.cn (M.R.); jlsun@shu.edu.cn (J.S.); 2 Shanghai Collaborative Innovation Center for High Performance Fiber Composites, Center for Civil Aviation Composites, Donghua University, Shanghai 201620, China; lyzhang@dhu.edu.cn

**Keywords:** SCNTs, conductive layer, antistatic agent, fiber

## Abstract

The hybrid antistatic agent SCNTs/OAA composed of sulfonated carbon nanotubes (SCNTs) and organic antistatic agent (OAA) was treated on the fiber surface to construct the hybrid conductive layer. Among them, SCNTs were synthesized through a simple method, and their chemical structure and morphology were characterized. SCNTs had good dispersibility due to the presence of sulfonic acid groups, which made SCNTs uniformly dispersed on the fiber surface. The SCNTs/OAA-treated fiber was hardly affected by relative humidity, because SCNTs form a continuous and uniform physical conductive network on the fiber surface. When the addition amount of SCNTs/OAA was 0.5~2 wt%, the fiber had excellent antistatic ability. Under the synergistic effect of SCNTs and OAA, the resistivity of SCNTs/OAA-treated fiber was almost not affected by fiber stretching.

## 1. Introduction

In the textile industry, a large number of static charges accumulated between fibers bring serious problems in the fiber nonwoven field. Therefore, it is desirable to construct a conductive layer on the fiber surface by adding antistatic agents. Generally, the antistatic agents are molecules containing hydrophilic and lipophilic groups. The lipophilic groups are adsorbed on the surface of the materials, while the hydrophilic groups are arranged towards the air side to absorb water molecules in the environment. These water molecules form a monomolecular conductive layer that can reduce surface resistivity [1,2,3]. Different antistatic agents were prepared based on this principle. Gürakın et al. [4] reported that the resistivity of polyethylene terephthalate dropped from 10^15^ to 10^9^ Ω⋅cm when 15 wt% of polyester-ether copolymer was added as an antistatic agent. Considering the cost, antistatic effect and application situation, the additional amount of antistatic agent usually does not exceed 5 wt%, but in this report, the addition amount of antistatic agent reached 15 wt%, so the addition amount was considered too large. Gao [5] and Kugimoto et al. [6] prepared antistatic agents containing quaternary ammonium salt and applied them to epoxy resin and polymethyl acrylate, respectively. The results showed that their resistivity decreased significantly with the increase of humidity. Based on the above works, it was found that traditional antistatic agents had some disadvantages such as large amounts of addition, being greatly affected by humidity, and limited antistatic effect.

Replacing these traditional antistatic agents with conductive fillers such as carbon black [7,8,9], glassy carbon [10,11], metallic oxides [12,13], and conductive polymer [14] can improve conductivity and stability. These conductive fillers can form a physical conductive network to dissipate the charge, so the stability of the antistatic effect is achieved [15]. However, their disadvantages are also obvious. For example, the addition of carbon black is large, usually more than 10 wt%; the antistatic effect of glassy carbon is limited; the preparation method for doping modification of metallic oxides and conductive polymers is complex.

In recent years, researchers have found that some nanomaterials, such as graphene, can also be used as conductive fillers, with some research results [16,17,18,19]. However, the high cost of preparation and the difficulty of industrialization hinder the large-scale application of graphene. Carbon nanotubes (CNTs), as a promising nano-conductive filler, have an efficient network-like conducting structure, high chemical stability, and remarkable mechanical properties [20,21]. Furthermore, the mature and cheaper preparation method, as well as the modification processes, made CNTs widely used for the modification of antistatic materials in the fields of fabric [22,23,24] and polymeric matrices [25,26,27]. However, it should be pointed out that in these works, simply adding CNTs to improve the antistatic ability is not enough. Because its conductivity is affected by many factors, such as agglomeration and addition amount, thereby limiting the improvement of the antistatic effect.

In order to solve the above-mentioned problems, a hybrid antistatic agent composed of organic antistatic agent (OAA) and CNTs was designed. Taking polyacrylonitrile-based pre-oxidized fiber (hereinafter referred to as “fiber”) as an example, the hybrid antistatic agent was applied to its surface to form the hybrid conductive layer. To make CNTs uniformly dispersed on the fiber surface, sulfonated CNTs (SCNTs) were synthesized. Then, the effect and mechanism of relative humidity, antistatic agent content, and fiber elongation on the antistatic ability of the hybrid antistatic agent were investigated.

## 2. Experimental

### 2.1. Materials

Carboxyl-functionalized multiwall carbon nanotubes (CCNTs, purity > 98%) were provided from Chengdu Organic Chemicals Co., Ltd. (Chengdu, China). *N*,*N*′-dicyclohexylcarbodiimide (DCC, analytically pure), 4-aminobenzene sulfonic acid (ACS, analytical pure), and anhydrous ethanol (analytically pure) were supplied by Sinopharm Chemical Reagent Co., Ltd. (Shanghai, China). 2-pentanol, 1,1′,1″,1‴-(1,2-ethandiyldinitrilo) tetrakis, and polyoxyethylene fatty acid were provided by Linyi Lvsen Chemical Co., Ltd. (Linyi, China). Primary alcobol ethoxylate was supplied by BASF (China) Co., Ltd. (Shanghai, China). Hexadecyl trimethyl ammonium bromide and alkapolpeg-400 were supplied by Sinopharm Chemical Reagent Co., Ltd. (Shanghai, China).

### 2.2. Synthesis of SCNTs

**Figure 1:**
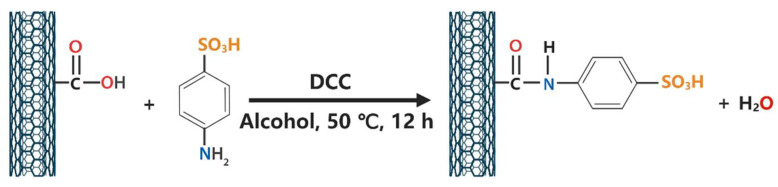
Synthesis procedure of SCNTs.

First, 1 g of CCNTs was dissolved into 250 g of anhydrous ethanol solution containing 1.7 g of DCC, and the dispersion solution was obtained by ultrasonication at room temperature for 35 min at the power of 560 W and frequency of 40 kHz. Then, 250 g of deionized aqueous solutions containing 1 g of ACS were then mixed with the dispersion solution, and stirred at 50 °C for 12 h. The mixed solution was vacuumed and filtered to obtain crude product. The crude product was washed alternately with anhydrous ethanol and deionized water until the pH of the filtrate was close to 7.0, followed by being dried under vacuum at 90 °C to obtain SCNTs. The synthesis process is shown in Figure 1.

### 2.3. Preparation and Application of Hybrid Antistatic Agent

**Figure 2:**
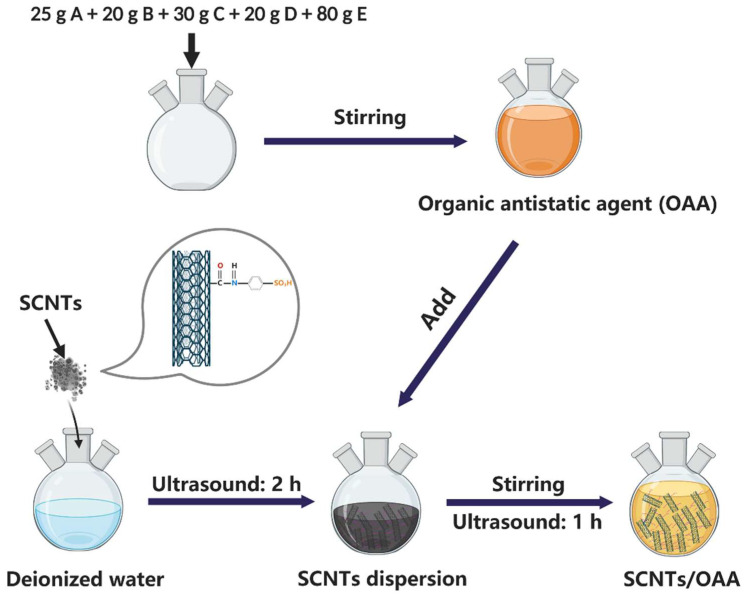
The preparation procedure of SCNTs/OAA.

The formulation of OAA is shown in Table 1 [28]. The preparation process of OAA and the hybrid antistatic agent SCNTs/OAA are shown in Figure 2, in which the content of SCNTs was 0.5 wt%. The preparation of the hybrid antistatic agent CCNTs/OAA was the same as that of SCNTs/OAA illustrated in Figure 2. Then, the OAA, CCNTs/OAA, and SCNTs/OAA with the content of 0, 0.5, 1.0, 1.5, 2, and 3 wt% were uniformly treated on the fiber surface by self-designed pretreatment process. After that, these fibers were placed in the air for 24 h to allow the antistatic agent to penetrate the fiber surface. Finally, the antistatic fiber was obtained. The antistatic fiber can be applied to the nonwoven process such as opening and netting, and finally to produce the integrated felts for C/C composite materials.

### 2.4. Characterizations and Testing

The functional groups on the surface of CNTs were investigated using Fourier transform infrared spectrometer (FT-IR, Nicolet iS10, Midland, ON, Canada), which was measured as pellets with KBr. The thermogravimetric analysis (TGA, STA 2500, Selb, Germany) of CNTs was conducted from 25 to 800 °C at 10 °C/min under nitrogen atmosphere. The morphology of CNTs was observed using transmission electron microscope (TEM, JEM-2010F, Newark, DE, USA), and the samples were obtained by dropping the ultrasonicated mixture of CNTs and ethanol onto a copper grid and allowed it dry in the air. The particle size distribution was measured using the laser particle size analyzer (Mastersizer 3000, Malvern, UK).

The electrical resistivity of fiber was measured using a fiber specific resistance meter (YG321D, Changzhou Huafang Textile Instrument Co., Ltd., Changzhou, China), refer to FZ/T 50035-2016 (National textile industry-standard of China). The specific steps of the resistivity test method under different relative humidity were as follows: First, the fiber specific resistance meter was placed in a room of 25 °C, and then the fiber was placed in a humidity chamber (KB-TH-S-80Z, Guangdong Kebao Test Equipment Co., Ltd., Dongguan, China). The temperature of the humidity chamber was fixed at 25 °C and the humidity was adjusted to the corresponding value. After placing for 1 h, the fiber was quickly removed for testing. The test was completed at about 10 s, and the data were obtained by repeated several times. The specific steps of the resistivity test method under different elongation were as follows: The two ends of the fiber were fixed on the specific resistance meter, and then on one end of the fiber, tensile action was gradually applied to measure its resistivity under different elongation. The optical microscope (HIROX, RH-2000) was used to directly measure the contact angle of the fiber. Specific steps were as follows: The fibers were suspended under a microscope, then water and ethylene glycol were dripped onto the fiber surface, respectively; then, take multiple photos continuously, the contact angle was measured by the software. Scanning electron microscope (SEM, Regulus-8100) was employed to observe the surface morphology of the fiber.

## 3. Results and Discussion

### 3.1. Characterizations of SCNTs

**Figure 3:**
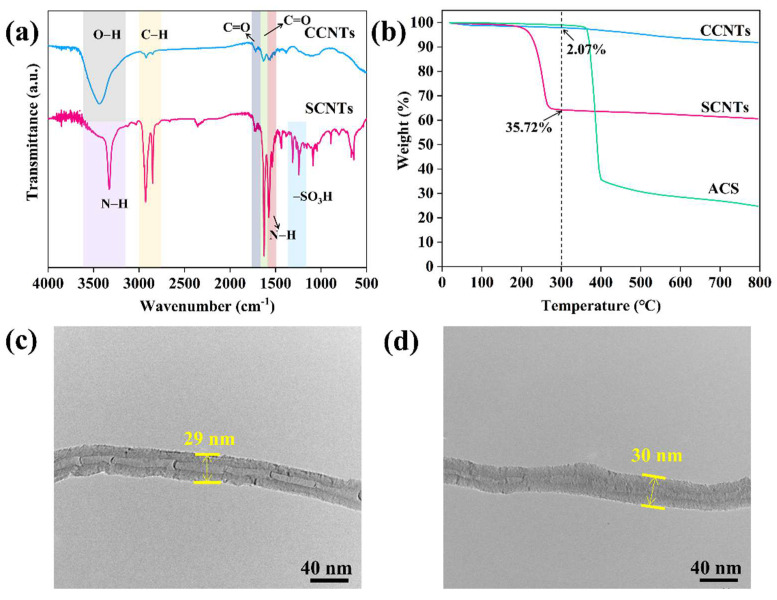
(**a**) FTIR spectra (the values were shifted vertically for clarity) and (**b**) TGA curves of CCNTs and SCNTs; TEM images of (**c**) CCNTs and (**d**) SCNTs.

Figure 3a shows the FTIR spectra of CCNTs and SCNTs. For CCNTs, the peak at 1730 cm^−1^ was attributed to characteristic C=O stretching vibration of carboxyl. The broad absorption at 3435 cm^−1^ was attributed to the O–H stretching vibration of hydroxyl groups. After it reacted with ACS, the strong peak at 1626 cm^−1^ of the C=O stretching vibration of amide, and two peaks at 1574 and 3326 cm^−1^, representing the characteristic peaks of N-H vibration of amino groups, were detected in SCNTs. Meanwhile, the absorption peaks at 1243 and 1311 cm^−1^ represent sulfonic acid groups. This observation indicated that SCNTs were successfully synthesized. The weight ratio of sulfonic acid groups on SCNTs can be calculated from the curve of TGA. As shown in Figure 3b, the decomposition temperature of ACS was around 350 °C. Therefore, the weight loss of SCNTs at around 200 °C was not caused by the residual monomer ACS, but by the decomposition of the grafted groups on the surface. The weight loss fraction of CCNTs and SCNTs were 2.07and 35.72 wt%, respectively, at 300 °C. Thus, the weight fraction of groups grafted onto the SCNTs can be estimated using the following equation:

(1) b%=(1−X)×a%+X

where a% and b% are the weight loss percent of CCNTs and SCNTs at 300 °C, respectively, and X represents the weight fraction of groups grafted onto the SCNTs. The content of groups grafted onto the SCNTs was calculated as 34.4 wt%. According to the further calculation of the relative molecular mass, the content of sulfonic acid group was about 14 wt%.

The surface morphologies of CCNTs and SCNTs were investigated using TEM. As can be seen from Figure 3c,d, the surface morphology of SCNTs remained smooth and clear compared with CCNTs, implying that no new defects were generated during the grafting process.

**Figure 4:**
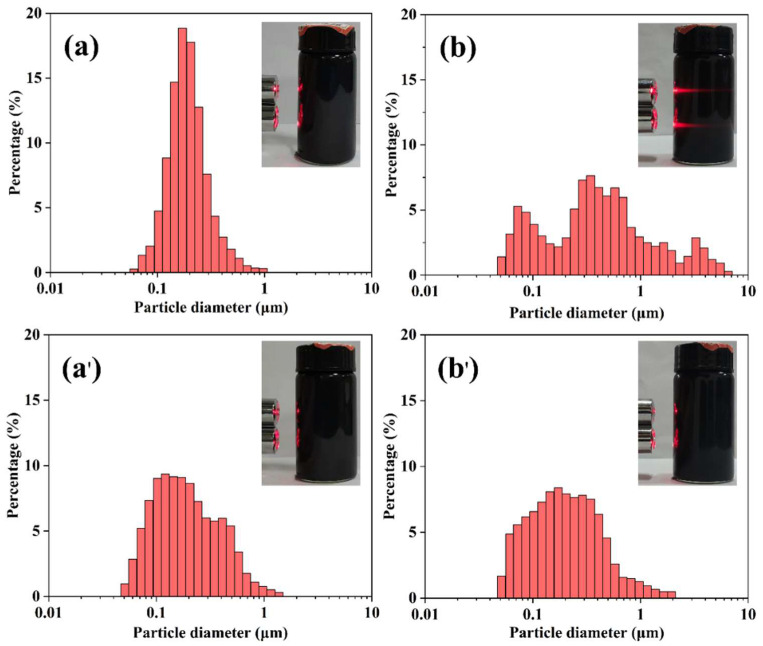
The particle size distribution of 0.2 wt% CCNTs (**a**,**b**) and 0.2 wt% SCNTs (**a’**,**b’**) in water (**a** and **b** refer to 1 min and 24 h, respectively, the embedded graphs were the corresponding dispersity photographs).

Then, to investigate the effect of the sulfonic acid groups on the dispersion of CNTs, CCNTs and SCNTs were dispersed in water at a ratio of 0.2 wt%, respectively. The embedded graphs in Figure 4 showed the dispersity photographs of CCNTs and SCNTs in water solution. After placing CCNTs for 24 h, the laser completely penetrated the solution. The results showed CCNTs agglomerated and drop to the bottom of the bottle after 24 h, while SCNTs did not. This indicated that SCNTs had rather good dispersibility. Because the agglomeration of particles depended on the particle size, the laser particle size analyzer was used to test the particle size of the dispersion at different time periods. The average particle size of CCNTs was 0.19 μm (Figure 4a) and 0.72 μm (Figure 4b) at 1 min and 24 h, respectively, and the particle size distribution became wider. Correspondingly, the average particle size of SCNTs was 0.22 μm (Figure 4a’) and 0.25 μm (Figure 4b’), respectively, and the particle size distribution hardly changed. It was consistent with the results obtained from the dispersion photographs. All these indicated that SCNTs had better dispersibility and stability in water. This excellent dispersion effect and stability were of great significance to the effect and stability of hybrid antistatic agent.

### 3.2. Mechanism Analysis of Antistatic on Fiber Surface

In order to explore the effect of relative humidity and antistatic agent content on fiber antistatic ability, the resistivity of the fiber under different relative humidity and antistatic agent content was characterized, respectively.

**Figure 5:**
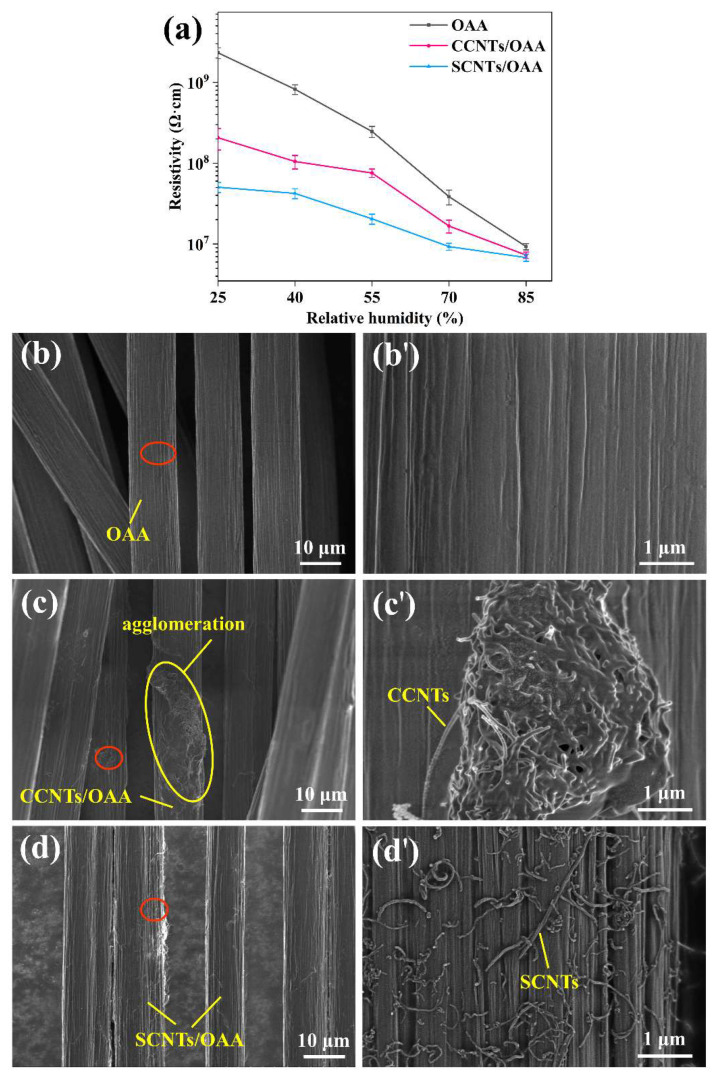
(**a**) Resistivity of fiber under different relative humidity; SEM images of fiber treated with (**b**) OAA, (**c**) CCNTs/OAA, and (**d**) SCNTs/OAA. (**b’**–**d’**) The enlarged images in the red circle.

Figure 5a shows the resistivity of fiber treated with OAA, CCNTs/OAA, and SCNTs/OAA under different relative humidity, respectively. When the relative humidity reduced from 85% to 25%, the resistivity of OAA-treated fiber changed dramatically by nearly 250 times. In comparison, for CCNTs/OAA-treated fiber and SCNTs/OAA-treated fiber, their resistivities changed by 28 times and 7 times, respectively. It is obvious that SCNTs/OAA-treated fiber was least affected by relative humidity than OAA-treated fiber and CCNTs/OAA-treated fiber. Moreover, the antistatic agents designed by Gao [5] and Kugimoto [6] was greatly affected by humidity, and the resistivity changed by 2~3 orders of magnitude. Therefore, SCNTs/OAA had good stability under low relative humidity. This was attributed to that when the relative humidity was low, the hydrophilic groups in OAA cannot absorb water molecules to form a conductive layer, but the physical conductive network formed by CCNTs and SCNTs played a role. According to Figure 5c’,d’, the diameter of CCNTs and SCNTs was about 30 nm, which was consistent with the TEM test results. This result further confirmed the presence of CNTs on the fiber surface. In addition, CCNTs form an agglomerated network (as shown in Figure 5c,c’), while SCNTs form a continuous and uniform conductive network (as shown in Figure 5d,d’), so SCNTs have more efficient conductive effects. The reason for the agglomeration was that the electrostatic repulsion between the carboxyl anions on the CCNTs surface was not capable of overcoming the attraction of van der Waals force between CCNTs. After functionalization with sulfonic acid groups, the electrostatic repulsion between SCNTs increased significantly, which was conducive to the dispersion of SCNTs on the fiber surface [29].

**Figure 6:**
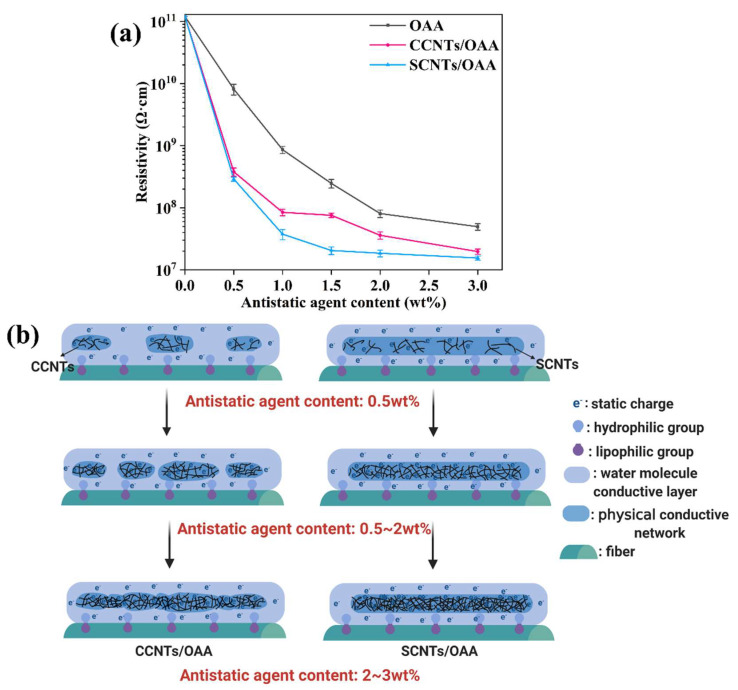
(**a**) Resistivity and (**b**) antistatic mechanism diagram of fiber with different antistatic agent content.

The antistatic agents with different contents were treated on the fiber surface, and the resistivity of fiber was tested. The result in Figure 6a showed that as antistatic agent content increased, the resistivity of fiber gradually decreased. The resistivity of fiber further reduced if OAA was substituted by CCNTs/OAA and SCNTs/OAA. It demonstrated that the synergistic conductive effect of CNTs and OAA further promoted the dissipation of static charges. Furthermore, Figure 6a also shows that when antistatic agent content reached 0.5 wt%, the resistivities between CNTs-added fibers were similar. When antistatic agent content was 0.5~2 wt%, the difference of resistivities between fibers became obvious. In particular, when the antistatic agent content was 1.5 wt%, the resistivity of SCNTs/OAA-treated fiber (2.05 × 10^7^ Ω⋅cm) was 73% lower than that of CCNTs/OAA-treated fiber (7.57 × 10^7^ Ω⋅cm). As antistatic agent content exceeded 2 wt%, the resistivities of fiber tend to be stable.

The related antistatic mechanism diagram is shown in Figure 6b. When the antistatic agent content was 0.5 wt%, there were not abundant CNTs to form a continuous conductive network, resulting in no difference in resistivity. When the antistatic agent content continued to increase to 0.5~2 wt%, the agglomerated CCNTs cannot form a continuous conductive network, so the resistivity of CCNTs/OAA-treated fiber did not decrease much. However, the uniform and continuous conductive network formed by SCNTs played a role, thus the resistivity of SCNTs/OAA-treated fiber decreased significantly. When antistatic agent content exceeded 2 wt%, a large number of CNTs made the resistivity of fiber reached a threshold, so the resistivity tended to be stable. Therefore, under the appropriate amount of addition, the good dispersion effect of SCNTs will show high-efficiency conductivity.

### 3.3. Effects of Fiber Stretching on Conductive Layer and Mechanism Analysis

During the nonwoven process such as opening and netting, the antistatic ability could be affected during the stretching between crimped fibers. Hence, the effect of fiber stretching on the conductive layer was studied. Combined with the practical application, the fiber with 1.5 wt% antistatic agent content was selected here for testing.

**Figure 7:**
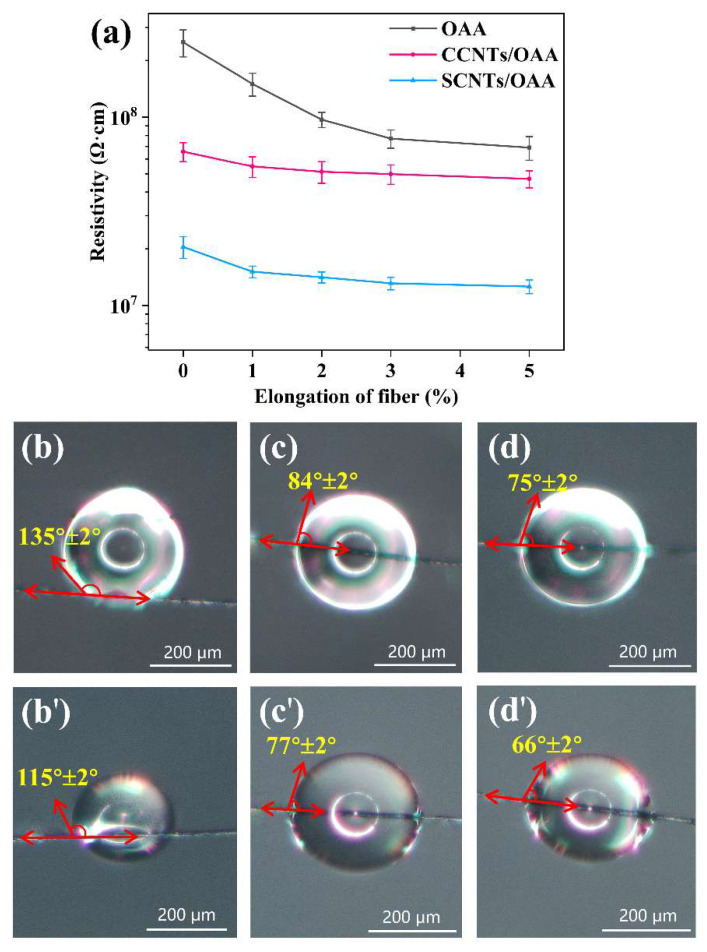
(**a**) Resistivity of fiber with different elongation; the contact angle of (**b**,**b’**) OAA-treated fiber, (**c**,**c’**) CCNTs/OAA-treated fiber, and (**d**,**d’**) SCNTs/OAA-treated fiber with water (**b**,**c**,**d**) and ethylene glycol (**b’**,**c’**,**d’**), respectively.

**Figure 8:**
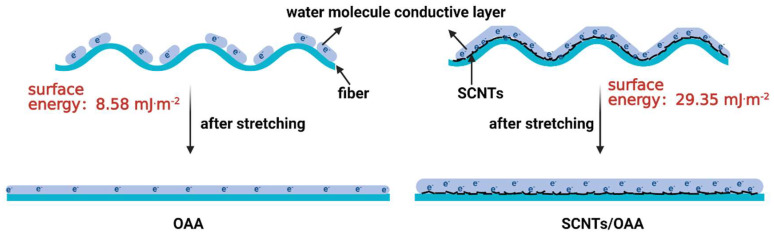
Antistatic mechanism diagram during the stretching process.

Figure 7a shows resistivities of fiber under different elongation. For OAA-treated fiber, the resistivity decreased sharply with the increase of elongation. For CCNTs/OAA-treated fiber and SCNTs/OAA-treated fiber, as the elongation increased, their resistivity only had a slight trend of decline. It was observed that the SCNTs/OAA-treated fiber surface was hydrophilic. To explain this phenomenon, the contact angle and surface energy ($\gamma_{S}$), including the polar component ($\gamma_{S}^{p}$) and the dispersive component ($\gamma_{S}^{d}$) of fiber samples, were studied. The result of contact angle is shown in Figure 7b–d. In this work, the method from Young [30], Fowkes [31], and Owens and Wendt [32] was applied for measuring the surface energy of fiber. The calculation formula of surface energy was as follows:

(2) $$\gamma_{L}\left(1+cos\theta \right)=2\sqrt{\gamma_{L}^{d}\gamma_{S}^{d}}+2\sqrt{\gamma_{L}^{p}\gamma_{S}^{p}}$$

(3) $$\gamma_{S}=\gamma_{S}^{p}+\gamma_{S}^{d}$$

where θ is the measured contact angle of fiber; $\gamma_{L}$, $\gamma_{L}^{d}$, and $\gamma_{L}^{p}$ have to be known for the test liquids used to contact the surface. Here, water and ethylene glycol were chosen for testing. The test results are shown in Table 2. The results indicated that SCNTs/OAA improved the surface energy of the fiber, which was attributed to the synergistic effect of the polar groups in OAA and sulfonic acid groups in SCNTs. The related mechanism diagram is shown in Figure 8. For OAA-treated fiber, the low surface energy made it more difficult to form a continuous water molecule conductive layer on the crimped fiber surface. When OAA-treated fiber was stretched, the originally disconnected water molecules became a continuous thin conductive layer, which significantly reduced resistivity. On the contrary, SCNTs/OAA-treated fiber had high surface energy, which lead to the formation of a continuous water molecule conductive layer on the crimped fiber surface. Meanwhile, the physical conductive network formed by SCNTs was not affected during the stretching process. Therefore, under the synergistic effect of OAA and SCNTs, the resistivity of SCNTs/OAA-treated fiber was almost unaffected by fiber stretching. The mechanism of CCNTs/OAA-treated fiber was consistent with this.

## 4. Conclusions

In this paper, the hybrid conductive layer composed of SCNTs and OAA was constructed on the fiber surface. The dispersibility of SCNTs was better than that of CCNTs due to the presence of sulfonic acid groups, which made SCNTs form a continuous and uniform conductive network on the fiber surface. This reduced the resistivity of the fiber and improved its antistatic ability, so the fiber was hardly affected by relative humidity. The optimal addition amount of SCNTs/OAA was 0.5~2 wt%, because the good dispersion effect of SCNTs at this time showed high-efficiency conductivity. Meanwhile, under the action of fiber stretching, the resistivity of the fiber in the crimped state can remain stable due to the synergistic effect of SCNTs and OAA. Therefore, the hybrid antistatic agent SCNTs/OAA was of great significance to the fiber nonwoven field.

## Figures and Tables

**Table 1:** The formulation of OAA.

Ingredient	Serial Number	Masses
2-pentanol, 1,1′,1″,1‴-(1,2-ethandiyldinitrilo) tetrakis	A	25 g
polyoxyethylene fatty acid	B	20 g
primary alcobol ethoxylate	C	30 g
hexadecyl trimethyl ammonium bromide	D	20 g
alkapolpeg-400	E	80 g

**Table 2:** The surface energy of fibers.

Sample	OAA-Treated Fiber	CCNTs/OAA-Treated Fiber	SCNTs/OAA-Treated Fiber
$\gamma_{S}$ (mJ·m^−2^)	8.58	22.85	29.35
$\gamma_{S}^{p}$ (mJ·m^−2^)	8.42	19.23	23.86
$\gamma_{S}^{d}$ (mJ·m^−2^)	0.16	3.62	5.49

## Data Availability

The data presented in this study are available on request from the corresponding author.
